# Cohort profile: the Diet and Healthy Aging (DaHA) study in Singapore

**DOI:** 10.18632/aging.104051

**Published:** 2020-11-18

**Authors:** Rongjun Yu, Ye Sun, Kaisy Xinhong Ye, Qiushi Feng, Su Lin Lim, Rathi Mahendran, Irwin Kee-Mun Cheah, Roger Sik Yin Foo, Ru Yuan Chua, Xinyi Gwee, Marie Loh, Rani Sarmugam, Wei Wei Thwe Khine, Yin Xia Chao, Anis Larbi, Yuan Kun Lee, Alan Prem Kumar, Brian K. Kennedy, Ee Heok Kua, Lei Feng

**Affiliations:** 1 Department of Psychology, Faculty of Arts and Social Sciences, National University of Singapore, Singapore, Singapore; 2 Department of Psychological Medicine, Yong Loo Lin School of Medicine, National University of Singapore, Singapore, Singapore; 3 Department of Sociology, National University of Singapore, Singapore, Singapore; 4 Department of Dietetics, National University Hospital, Singapore, Singapore; 5 Department of Psychological Medicine, National University Hospital, Singapore, Singapore; 6 Academic Development Department, Duke-NUS Medical School, Singapore, Singapore; 7 Department of Biochemistry, Yong Loo Lin School of Medicine, National University of Singapore, Singapore, Singapore; 8 Cardiovascular Research Institute, Yong Loo Lin School of Medicine, National University of Singapore, Singapore, Singapore; 9 Lee Kong Chian School of Medicine, Nanyang Technological University, Singapore, Singapore; 10 Department of Epidemiology and Biostatistics, Imperial College London, London, United Kingdom; 11 Health Promotion Board, Singapore, Singapore; 12 Department of Microbiology and Immunology, Yong Loo Lin School of Medicine, National University of Singapore, Singapore, Singapore; 13 Department of Neurology, National Neuroscience Institute, Singapore, Singapore; 14 Department of Medical Education, Research and Evaluation (MERE), Duke-NUS Medical School, Singapore, Singapore; 15 Singapore Immunology Network, Agency for Science, Technology and Research (A*STAR), Singapore, Singapore; 16 Cancer Science Institute of Singapore, National University of Singapore, Singapore, Singapore; 17 Department of Pharmacology, Yong Loo Lin School of Medicine, National University of Singapore, Singapore, Singapore; 18 Department of Physiology, National University Singapore, Singapore, Singapore; 19 Singapore Institute of Clinical Sciences, Agency for Science, Technology and Research (A*STAR), Singapore, Singapore; 20 Centre for Healthy Longevity, National University Health System, Singapore, Singapore

**Keywords:** cognitive impairment, DaHA, diet, cohort profile, healthy ageing

## Abstract

How diet is related with cognition and health has not been systematically examined in Asians whose eating habits are very different from their counterparts in the West and the biological mechanisms underlying such links are not well known yet. The diet and healthy aging (DaHA) study is a community-based longitudinal study conducted to examine the role of diet and nutrition in promoting cognitive, emotional, and physical health among community-living elderly Singaporeans. The first wave of DaHA, conducted from 2011 to 2017, provided detailed information on diet and baseline cognitive function and health from 1010 community-living elderly in Singapore. Biomarkers of oxidative stress, systemic inflammation, and genetic information were collected. The ongoing second wave of DaHA is conducted from 2017 to 2020, which provides follow- up assessments using established cognitive tests and clinical tools. This well-characterized cohort, with its archived biological samples and high-quality data on diet and lifestyle factors will allow researchers to explore the relationships among diet, nutrition, genes, cognition, mental and physical health in an extremely cost-effective manner. Translations of the research findings into clinical and public health practices will potentially help to promote cognitive health at the population level and reduce healthcare costs related to cognitive impairment.

## INTRODUCTION

The latest nationwide epidemiological survey in Singapore reported that the prevalence of dementia was 10% in the older adult population (aged 60 years and above) based on the 10/66 diagnosis of dementia, while the prevalence of Diagnostic and Statistical Manual of Mental Disorders, 4^th^ Edition (DSM-IV), a more restrictive criteria, dementia was 4.6% [1]. This figure is expected to rise further in the coming years given the rapidly aging population in Singapore. The worldwide costs of dementia are estimated to be US$818 billion in 2015, which is approximately 1% of the world’s gross domestic product [2].

How good cognitive health in the later stage of life can be successfully maintained, is an important and challenging question that requires well-planned research with follow-up translational opportunities. Potential avenues for interventions must be identified from carefully designed longitudinal studies to establish cause and effect relationships. The value of such studies lies in firmly establishing important modifiable risk and protective factors that could be targeted in future preventive interventions. Prevention requires a good understanding of the natural history of the condition and a complete and comprehensive picture of various risk and protective factors and biological markers that could be involved in the process. This information can only be obtained from population-based longitudinal cohort studies [3]. Moreover, the importance of prevention lies in the fact that none of the currently approved drug therapies can reverse the disease progression of dementia. The condition of patients who take these drugs remains stable for a year or more but declines subsequently, although at a rate that is slower than that among untreated patients [4]. The search for a disease-modifying agent has proven to be equally elusive. Results from recent large-scale phase 3 trials involving disease-modifying agents such as the anti-amyloid-β monoclonal antibody drugs solanezumab and bapineuzumab were essentially negative [5, 6]. The disappointing results from these studies have provided the impetus for the increasing shift in attention to non-pharmacological preventive lifestyle measures such as diet.

### Diet and cognition

In the West, several studies have reported the association between dietary patterns and cognitive status. For example, the Mediterranean diet, a dietary pattern usually consumed among the populations bordering the Mediterranean Sea, has been reported to be protective against Alzheimer’s disease (AD) and cognitive decline [7, 8]. More recently, Morris and colleagues investigated the “diet and Alzheimer’s disease” relationship in a prospective cohort study of 923 participants, aged 58 to 98 years, followed up for an average of 4.5 years [9]. Three diets were studied, and the researchers found that certain diets were associated with a reduced risk of dementia. However, these dietary patterns and other known brain-healthy dietary patterns were all developed based on the eating habits of Caucasian populations and may not apply to elderly populations in Asia. Recent publication on dietary pattern and cognitive impairment in Singapore Chinese showed that adherence to healthy dietary patterns in midlife is associated with a lower risk of cognitive impairment in late life [10]. Preliminary data from interventional trials support further research on diet and cognitive health. In one small trial, Bayer-Carter and colleagues reported favourable changes in cerebrospinal fluid biomarker profiles after manipulating the intake of saturated fat and simple carbohydrates [11]. In another study, improvements in the function of the dentate gyrus were shown among trial participants who consumed a high cocoa-flavanol diet for three months [12]. Since flavanols are found naturally in tea leaves and in certain fruits and vegetables, the trial data indirectly support the cognitive benefits of consuming such dietary items.

The neuroprotective effects of certain dietary factors and patterns may be due to their antioxidant and anti-inflammatory properties. It is well known that the human brain is highly susceptible to oxidative damage because of its high metabolic load and its abundance of oxidizable material. The major free radicals such as superoxide anion radicals, hydroxyl radicals and nitric oxide have been implicated in brain ageing and cognitive impairment while oxidative stress is recognized as one of the fundamental mechanisms across the spectrum of cognitive performance [13]. Human ageing is also characterized by a chronic, low-grade inflammation, termed as "inflammaging" [14]. Inflammaging is a significant risk factor for both morbidity and mortality and most, if not all, age-related diseases share an inflammatory pathogenesis. Both oxidative and inflammatory damages have been well documented in the disease pathophysiology of AD [15]. Many dietary components (for example, polyphenols and ergothioneine from plant-derived foods, curcumin from curry, long chain omega-3 polyunsaturated fatty acids from fish) have antioxidant and anti-inflammatory properties. Thus, the hypothesis that diet affects cognition through reduced oxidative damage and inflammation has a valid biological basis and is likely to be confirmed by the current study with a rigorously designed measurement plan.

### *APOE* and *FOXO*

Genetic polymorphism may also mediate how diet influences cognition and health. It has been found that genetic variations within the *APOE* and *FOXO* genes are strongly associated with human longevity [16, 17]. Apolipoprotein E (*APOE*) gene encodes for a polymorphic 299-amino acid protein which has critical functions in redistributing lipids among central nervous system (CNS) cells, repairing injured neurons, maintaining synapto-dendritic connections, and scavenging toxins [18]. The *APOE*-epsilon 4 (*APOE* ε4) allele is a well-known major genetic risk factor for AD. Based on functional Magnetic Resonance Imaging (MRI) studies with subjects in their 20s and 30s, the *APOE* ε4 allele modulates brain function decades before any clinical expression of neurodegenerative processes, and the effects are not explained by differences in memory performance, brain morphology, or resting cerebral blood flow [19]. Neuroimaging studies have indicated that in non-demented individuals, the *APOE* ε4 genotype is associated with structural and functional brain changes such as greater hippocampal volume loss and reduced brain white matter integrity [20, 21]. In Singapore, we have reported that the proportion of *APOE* ε4 carriers is 16% among those aged 55 and above [22]. The large number of individuals who are carrying this genetic risk factor represents an important subpopulation that requires targeted and evidence-based early interventions.

Forkhead box O (*FOXO*) transcription factors, *FOXO1A* and *FOXO3A*, have been found associated with longevity in Han Chinese [23, 24]. These transcription factors are known to regulate pathways associated with longevity via insulin and insulin-like growth factor signalling [25]. Insulin-like growth factors (IGFs) have been suggested as important modifiers for the pathogenesis of neurodegenerative diseases in ageing [26]. Hence, *FOXO1A* and *FOXO3A* carriers are said to be protected against age-related cognitive decline.

Although the main effect of *APOE* and *FOXO* on brain ageing has been widely studied and has yielded interesting findings, the role of these genes in modulating the relationships between multiple dietary factors and cognitive outcomes remains unknown.

### Study objectives

The diet and healthy aging (DaHA) study was setup to address three specific aims. First, we aimed to identify dietary factors that are associated with better cognitive function, better mental and physical health, and reduced incidence of mild and major neurocognitive disorders. Our study may identify novel dietary patterns based on the eating habits of local seniors. Such work may potentially lead to the development of a useful diet research tool and add an Asian diet to the current list of established preventive diets such as the Mediterranean diet. Establishing longitudinal relationships between dietary factors and health outcomes will equip local practitioners with useful knowledge for them to provide dietary advice to their patients and clients confidently.

Second, we aimed to investigate if cognitive benefits of such dietary factors can be mediated partially by reduced oxidative damage and systemic chronic inflammation. For a deeper understanding of the biology between diet and cognition, we measured a number of biomarkers of oxidative stress and systemic inflammation to examine the potential mediating roles that they may play.

Third, we aimed to investigate the interaction between diet and gene polymorphism in influencing cognitive health in the elderly. For example, carriers of certain allelic polymorphisms of the *APOE* or *FOXO* genes may benefit more or less from healthy diets in cognitive health due to gene-nutrient interactions. Greater knowledge on the role of genes can lead to drug discovery that targets the potential causal molecular pathways implicated in the development of cognitive decline to improve health and quality of life in the elderly.

The study obtained approvals from the NUS Institutional Review Board [Reference number 10-517] and written informed consent from the participants.

## RESULTS

Only those who signed agreed consent from 2011 to 2017 were invited to the study centre for assessments. After removal of duplicates (n=6) and participants with missing entire data (n=44), one thousand and ten elderly persons aged 60 and above were recruited to participate in this population-based cohort, DaHA. 72% were females. Chinese (96.3%) accounted for the majority of the participants, followed by Indian (1.8%), then Malay (0.8%). The mean number of years of schooling among the participants was 6.3 ± 4.2.

There were 986 participants who provided complete information to be considered for the six criteria of “healthy ageing” (see Methods). 67 of them (6.8%) met all six criteria of “healthy ageing”, while the rest (n=919, 93.2%) were considered “normal ageing” (see Table 1). Among the “normal ageing” study participants, 84 (8.5%) rated themselves as not in good health; 684 (69.4%) suffered from major chronic diseases; 527 (53.4%) had poor physical function; 149 (15.1%) had poor cognitive function; 77 (7.8%) had non-optimal mental health status; and 366 (37.1%) did not have frequent social activities.

**Table 1:** Distribution of study participants with healthy or normal ageing in DaHA.

	**Normal ageing**		**Healthy ageing**		**Total**
	**N**	**%**	**N**	**%**	**N**
Overall	919	93.2%	67	6.8%	986
Self-rated health	84	8.5%	902	91.5%	986
Absence of disease	684	69.4%	302	30.6%	986
Physical function	527	53.4%	459	46.6%	986
Cognitive function	149	15.1%	837	84.9%	986
Emotional health	77	7.8%	909	92.2%	986
Social engagement	366	37.1%	620	62.9%	986

Several publications have been made from preliminary examinations of nutritional biomarkers, certain food items and dietary habits in relation to ageing, mental and cognitive health. Our cross-sectional data revealed the potential role of mushrooms and its bioactive compounds in delaying neurodegeneration. In our cohort, whole blood ergothioneine levels declined significantly beyond 60 years of age, and people with MCI had significantly lower plasma ergothioneine levels compared with age-matched subjects with normal cognitive function [27]. Mushrooms are one of the richest sources of ergothioneine. Examination of mushroom consumption found reverse association with the odds of having MCI. Participants who consumed mushrooms >2 portions per week had reduced odds of having MCI than those who consumed mushrooms less than once per week [28]. In examination of dietary habits, participants with frequent (≥4 days per week) fruit consumption and active (≥4 days per week) bowel movement within 10 minutes were negatively associated with MCI occurrence [29]. In addition, a cross-sectional evaluation of diet-mental health relationship found that long-term tea consumption was significantly associated with a reduced odds of having depressive and anxiety symptoms among DaHA participants [30]. In a Magnetic Resonance Imaging (MRI) study, we found that regular tea drinkers had more efficient brain structural network as compared with non-tea drinkers [31]. Those studies will be followed up by examining tea’s bioactive compounds such as L-theanine and catechins in the peripheral blood in cognitively impaired elderly subjects and cognitively normal subjects.

In a sub-study, targeted liquid chromatography-mass spectrometry analysis was conducted to quantify 49 oxylipins and 4 polyunsaturated fatty acids in the plasma samples of 60 subjects diagnosed with MCI and 56 age- and gender- matched cognitively normal individuals. Levels of linoleic acid (LA) and 7 oxylipins were significantly altered in MCI subjects. Notably, oxylipins synthesized through 5-lipoxygenase (5-LOX) and cytochrome P450 (CYP450) pathways of arachidonic acid (AA) or LA were elevated in MCI patients, suggesting an implication of inflammation in the etiology progression of cognitive impairment when ageing [32].

The habitual food consumption data obtained from the FFQ is currently being analysed to identify dietary patterns of the Singapore elderly population, which may provide protective or detrimental influences on the process of cognitive decline and ageing.

## DISCUSSION

The main strengths of the DaHA study include: (1) identifying the dietary factors that are associated with better cognitive outcomes in Asia. (2) investigation of the genetic polymorphisms and biomarkers of oxidative stress and systemic inflammation will help identify potential mediators in the relationships between diet and health and further clarify the underlying genetic and pharmacological mechanisms; (3) the multi-phase data collection provides a unique opportunity to understand the longitudinal changes of cognitive functions and gene-diet interactions; (4) our previous findings can be of great clinical and practical value, by informing on how to promote a healthy diet. Findings from this study have the potential to be developed into a dietary index that can be adopted by health service providers and community organizations as part of their health education and interventional programs. The translation of research findings into clinical and public health practice by the careful design and implementation of a dietary intervention program will lead to the reduction of incident cases of neurocognitive disorders at the population level and help to promote successful brain ageing among local seniors.

One limitation of this cohort design is that participants were recruited from a single site in western Singapore. However, given Singapore is a small city country, our cohort recruited from Jurong site near the shopping mall is quite representative of the Singaporean population. Moreover, the cohort effect may influence our results since the older adults in our study are the so-called “pioneer generation” in Singapore. Many older Singaporeans migrated to Singapore before it was an independent country in 1965. Thus, there are vast differences between our cohort in Singapore and cohorts in other Asian countries in terms of lifestyle and educational opportunities.

Nevertheless, this well-characterized cohort, with its archived blood samples and high-quality data on diet and lifestyle factors, represents an unprecedented opportunity to address the proposed aims in an extremely cost-effective manner.

## MATERIALS AND METHODS

We conducted door to door census to identify and invite eligible study participants from the Jurong area of Singapore. Inclusion criteria were community-living Singaporean or permanent residents aged 60 and above. Participants will be followed up every five years from baseline. The first follow up (second wave of DaHA) is conducted from 2017 to 2020; estimated rate of dropouts from this ongoing follow up is 30%.

Each subject had at least two sessions of assessment with the study research nurse or research assistant. The assessments were conducted at the Training and Research Academy at Jurong Point (TaRA@JP). The first session involved the collection of blood and urine samples, clinical measurements, questionnaire-based interview and cognitive tests. The second session consisted of another battery of cognitive measures and assessment tools. The assessment outcomes for mental and cognitive health from the first session were used to determine subject eligibility for the third session. Table 2 shows an overview of data measures collected at the baseline wave of DaHA.

**Table 2:** Overview of data measures collected at the baseline wave of DaHA.

**First session**	**Second session**	**Third session**	**Offline measure**
**Demographic** Age, gender, ethnics **Biological samples** Blood, urine, faeces **Physical health** Self-rated overall health, medical conditions, medications & supplements, blood pressure & pulse rates, body temperature, respiratory rate, weight, height, waist–hip ratio, handgrip strength and 6-meter walking speed test **Mental health** Geriatric depression scale (GD) and geriatric anxiety inventory (GAI) **Cognitive health** Singapore modified mini-mental state examination (SM-MMSE) and perceived deficits questionnaire (PDQ) **Diet patterns** Food frequency questionnaires (FFQ) and 3-day food record	**Physical health** Pittsburgh sleep quality index (PSQI) **Mental health** The global mental health assessment tool (GMHAT) **Cognitive health** Montreal cognitive assessment (MoCA), repeatable battery for the assessment of neuropsychological status (RBANS), and brief informant screening test (BIST)	**Neurocognitive assessment** Clinical dementia rating (CDR) Rey auditory verbal learning test (RAVLT) immediate recall Digit span Colour trails test (CTT) 1 & 2 Block design Rey auditory verbal learning test (RAVLT) delayed recall & recognition Verbal fluency test Symbol digit modalities test (SDMT) **Psychiatric assessment:** Structured Clinical Interview for DSM-IV Axis I Disorders (SCID-I)	**Bioactive compounds/Phytochemicals** Ergothioneine Catechins and L-theanine Curcumin OCTN1 **Oxidative damage markers** F2-IsoPs 8OHdG Allantoin & urate **Inflammatory and vascular markers:** TNF-a C-peptide VEGF-A PAI-1IP-10 sTNFR-1 sIL-2Rα C-reactive protein, Interleukin-1β, Interleukin-6 **Genes** *APOE*, telomere length, DNA methylation and RNAseq **Physiological and nutritional biomarkers** Full blood counts, lipoproteins, albumin, folate, vitamin B-12, homocysteine, fasting glucose, and creatinine

### Dietary assessments and analyses

Habitual dietary practices and intakes were measured using a validated dietary practices questionnaire (DPQ) and a food frequency questionnaire (FFQ) developed by the Singapore Health Promotion Board (HPB) [33]. The DPQ consists of 26 items covering place of dinning (e.g. home, eat out), food preferences (e.g. types of bread, rice, milk beverage, fat spread, sweetening agent), eating and cooking habits (e.g. removal of animal fats, and use of salts, sauces, fats and oils) and use of supplements and herbs. The FFQ was developed based on the eating habits of the local Singaporean. It contains more than 300 food items designed to capture the calories, total fat, types of fats, cholesterol, and other nutrients consumed by the subjects. The major food groups covered include grain foods, meat, poultry, fish and seafood, eggs, nuts, vegetables and tofu, fruits, milk and dairy products, soya products, vegetarian products, soups and discretionary food and drink choices. Subject responses were recorded as number of times the food is consumed per day, week or month. The food portions listed correspond to the numbers on the food models, which were used when asking subjects for the number of portions the food is normally consumed at one seating. Venues were asked for foods that could be eaten out or prepared at home, and cooking methods (e.g. steamed, stir-fried, curry without coconut) were captured to consider for the different nutrient compositions. Each subject also completed a 3-day food record (2 weekdays and 1 weekend day). The method of recording was explained to the subject by a trained research nurse. Illiterate subjects could record their food intake with helps from their family members or caregivers. The food record served two purposes: (1) to make correlation analyses to access the validity of FFQ; (2) to capture food items not listed in the FFQ. The intakes of various macro- and micronutrients were calculated based on the information obtained from the FFQ using a database system developed by the HPB. The database contains information on common locally consumed foods that are not seen in other countries.

In addition to the FFQ, we also collected detailed information on the consumption of vegetables and fruits using a long list of commonly consumed vegetables and fruits in Singapore. The dietary data collected will allow us to assess the associations between aging-related phenotypes and certain dietary factors such as mushrooms, vegetables, fruits, coffee, tea, sugary drinks, fish, red meat, soy foods, western-style fast food, micro- and macronutrients, and dietary patterns based on known patterns such as the Mediterranean diet and the Mediterranean – DASH Intervention for Neurodegenerative Delay (MIND) diet.

New dietary patterns will be derived from the FFQ data using Principle Component Analysis (PCA) [34]. Certain nutritional epidemiology specific analyses will be considered, such as adjustment for total energy, where applicable. At follow-up, we will use the repeated FFQ and biomarker measures as the exposures to study whether changes in eating habits and nutrient biomarkers, as continuous or categorical variables, are related to changes in cognition.

### Cognitive health assessments

Trained research nurses administered a modified Singapore version of the original Mini-Mental State Examination (SM-MMSE, range 0–30) to all subjects as a measure of cognitive function [35]. Participants who obtained a SM-MMSE score lower than pre-specified cut-off values (≤ 27 for subjects without formal education, ≤ 28 for primary school education level, ≤ 29 for secondary school and above) were invited for a third session for neurocognitive assessments; the assessment session consisted of history taking, Clinical Dementia Rating (CDR) and a battery of standard neuropsychological tests (see Table 2 for details) [36]. Completed clinical and psychometric data were reviewed by a panel that consisted of two senior consultant psychiatrists (EH Kua and R Mahendran), the PI (L Feng), and all cognitive assessors. The Petersen’s criteria of mild cognitive impairment (MCI) was used and objective cognitive impairment was determined using local norms (age and education adjusted) of the neuropsychological tests. Dementia was diagnosed based on the criteria in the DSM-IV.

### Mental health assessments

Participants who obtained a Geriatric Depression Scale (GDS) or Geriatric Anxiety Index (GAI) score greater than pre-specified cut-off values (≥3 for GDS, ≥5 for GAI) or a preliminary diagnosis from the Global Mental Health Assessment Tool (GMHAT) were also invited for a third session for psychiatric assessment using the Structured Clinical Interview for DSM-IV Axis I Disorders (SCID-I). The assessments were conducted by the Principal Investigator (PI) (L Feng) and two registrar psychiatrists. Completed cases were reviewed by two senior consultant psychiatrists (EH Kua and R Mahendran) together with the PI. Consensus diagnosis was made based on the DSM-IV criteria.

### Collection of biological samples

Blood and urine samples were collected to assay for biological markers that may provide scientific explanations for the expected benefits of certain foods, beverages, and dietary patterns. Collection of fasting venous blood samples was carried out by qualified research nurses. All participants who were eligible for dementia assessment or psychiatric assessment and a subgroup of participants who were not eligible for the assessments were invited for blood sample collection following standard venepuncture procedure.

Venous blood (13.5ml) was collected in K2-EDTA treated vacuum tubes (5.5 ml x2 tubes) and PAX gene blood tubes containing RNA stabilizing agents (2.5ml) for whole blood, plasma, lymphocytes (DNA), erythrocytes and RNA. Blood samples were kept at 4°C until they were processed by the research laboratory. Plasma was obtained by centrifugation of the EDTA blood tubes (within 2 hours of sample collection) at 2500 × g (for 15 min at 4°C) and then divided into 250 μl aliquots together with 2.5 μl of 2 mM BHT (as an antioxidant; prepared in ethanol) and 1 μl of 5 mM indomethacin (cyclooxygenase inhibitor). Lymphocytes and erythrocytes obtained from centrifugation of the EDTA blood tubes were divided into 250 μl aliquots and stored at -80°C. All aliquoted samples were coded and stored until required for analysis of biomarkers. A logbook was used to record detailed information on the number of storage tubes from each subject, the form of stored biological material for each tube, and corresponding subject numbers. An additional 10ml of fasting venous blood was collected for a subgroup of subjects for clinical laboratory tests to be undertaken at NUH referral laboratories. Blood was collected in K2-EDTA tubes (3ml), plain serum tubes (5ml) and sodium fluoride tubes (2ml) for full blood count, lipoproteins, homocysteine, albumin, folate, vitamin B12, creatinine and fasting glucose tests.

Urine samples (approximately 20ml) were collected in urine collection bottles and kept at room temperature. At the research laboratory, urine samples were separated into 2 ml aliquots (2 ml microcentrifuge tubes) and then stored at -80°C until required for biomarker analyses.

A subgroup of the participants was asked to collect a small amount of faeces (0.3 to 0.5g) that has been defecated on a trail paper, using a sterile fork. Sample was collected within 18 hours before the second visit and passed to the research nurse before the start of the session.

### Analysis of biological samples

Plasma from whole blood samples and urine were used for laboratory tests to examine the potential role of certain bioactive compounds, oxidative damage and inflammatory markers, physiological and nutritional biomarkers (see Table 2 for details) in the relationship between diet and cognitive health. Bioactive compounds were assessed using liquid-chromatography coupled with tandem mass spectrometry (LC-MS/MS). LC-MS/MS was also used to measure the level of F2-isoprostanes (F2-IsoPs), allantoin and urate in plasma and urine, and the level of 8-hydroxydeoxyguanosine (8OHdG) in urine samples. Allantoin levels were normalized to urate levels in the same sample. Pro-inflammatory cytokines were measured from plasma using Luminex assays. We used a pay-for-service at the National University Hospital Referral Laboratory for measurements of physiological biomarkers such as full blood counts and lipoproteins, and biomarkers of nutritional status.

Two *APOE* single nucleotide polymorphisms (SNPs) (rs429358 and rs7412) were genotyped from buffy coat samples to study its interactions with dietary and nutritional factors. Using the additional DNA extracted from buffy coat we plan to generate epigenome-wide methylation data. DNA methylome changes with increasing age. Recent studies have identified collections of individual methylation sites whose aggregate methylation status measures chronological age, referred to as the DNA methylation clock [37]. Given the modifiable nature of DNA methylome, we plan to see if there is an association between methylation age and dietary choices. We will use Illumina Infinium MethylationEPIC BeadChip, which covers over 850,000 methylation sites per sample at a single-CpG site resolution, and provides unparalleled coverage of CpG islands, RefSeq genes, ENCODE open chromatin, ENCODE transcription factor binding sites, and FANTOM5 enhancers, as well as regions identified by the ENCODE project as potential enhancers [38]. Subsequently, we will develop a methylation risk score (MRS) model by taking a weighted approach (by effect size) for the CpG loci identified to be associated with our endpoints. Using the information, we will calculate methylation age [39], as well as investigate the interactions between the identified epigenetic risk factors with environmental factors. Given the abundant blood samples collected, RNA sequencing and examination of telomere length are also under investigation.

### Sub-study

A subgroup of the participants was invited for brain Magnetic Resonance Imaging (MRI) scan. Image acquisitions were collected using a 32-channel head coil on a 3T Prisma Siemens MR scanner at the Clinical Imaging Research Centre (CIRC) at the National University of Singapore (NUS). High-resolution T1-weighted magnetization-prepared gradient-echo images, diffusion weighted images and functional MRI images were acquired.

### Defining “healthy ageing”

We examined the prevalence of healthy ageing among community-living Singaporeans using a working definition that covers a thorough spectrum of ageing aspects including self-rated health, absence of chronic diseases, physical and cognitive functioning, social engagement, and mental wellness [40, 41]. Although there is no universally accepted definition of healthy or successful aging, our working definition was based on the suggestion to focus on symptomatic disease and functional health outcomes rather than rigid disease criteria [41, 42]. To be specific, an elderly is considered to have achieved healthy ageing if he/she fulfils all of the following six criteria: (1) self-rated health: participants endorsed being in good to excellent heath (rating≥3) in a single item self-rated health measure [43]; (2) absence of chronic diseases: no self-reported medical history of 14 major chronic diseases, including hypertension, diabetes, stroke, heart attack, arrhythmias, heart failure, kidney failure, Chronic Obstructive Pulmonary Disease (COPD), arthritis, cancer, depression disorders, anxiety disorders, and other mental disorders; (3) physical function: the hand grip strength of the dominant hand was at or above the age- and gender- specific population mean minus one standard deviation using published norms of Singapore residents [44]; (4) cognitive function: no diagnosis of MCI or dementia after the thorough cognitive assessment procedures of DaHA, and with a Singapore modified mini-mental state examination (SM-MMSE) score of at least 26 (out of 30) points; (5) social engagement: the self-reported frequency of joining social activities is at least once per week; (6) mental wellness: no diagnosis of depressive disorders or anxiety disorders from the Structured Clinical Interview for DSM-IV (SCID-I) as part of DaHA mental health assessment protocol, and with a GDS score of less than 5 (out of 15) [45] and a GAI score of less than 9 (out of 20) [46].
